# Health disparities in the risk of severe acidosis: real-world evidence from the *All of Us* cohort

**DOI:** 10.1093/jamia/ocae256

**Published:** 2024-10-14

**Authors:** Allison E Gatz, Chenxi Xiong, Yao Chen, Shihui Jiang, Chi Mai Nguyen, Qianqian Song, Xiaochun Li, Pengyue Zhang, Michael T Eadon, Jing Su

**Affiliations:** Department of Medicine, Indiana University School of Medicine, Indianapolis, IN 46202, United States; Department of Biostatistics and Health Data Science, Indiana University School of Medicine, Indianapolis, IN 46202, United States; Department of Computer and Information Technology, Purdue University, West Lafayette, IN 47907, United States; Department of Biostatistics and Health Data Science, Indiana University School of Medicine, Indianapolis, IN 46202, United States; Department of Biostatistics and Health Data Science, Indiana University School of Medicine, Indianapolis, IN 46202, United States; Department of Biostatistics and Health Data Science, Indiana University School of Medicine, Indianapolis, IN 46202, United States; Department of Health Outcomes and Biomedical Informatics, College of Medicine, University of Florida, Gainesville, FL 32608, United States; Department of Biostatistics and Health Data Science, Indiana University School of Medicine, Indianapolis, IN 46202, United States; Department of Biostatistics and Health Data Science, Indiana University School of Medicine, Indianapolis, IN 46202, United States; Department of Medicine, Indiana University School of Medicine, Indianapolis, IN 46202, United States; Department of Biostatistics and Health Data Science, Indiana University School of Medicine, Indianapolis, IN 46202, United States

**Keywords:** health inequities, social determinants of health, electronic health records, acidosis, *All of Us*

## Abstract

**Objective:**

To assess the health disparities across social determinants of health (SDoH) domains for the risk of severe acidosis independent of demographical and clinical factors.

**Materials and Methods:**

A retrospective case-control study (*n* = 13 310, 1:4 matching) is performed using electronic health records (EHRs), SDoH surveys, and genomics data from the *All of Us* participants. The propensity score matching controls confounding effects due to EHR data availability. Conditional logistic regressions are used to estimate odds ratios describing associations between SDoHs and the risk of acidosis events, adjusted for demographic features, and clinical conditions.

**Results:**

Those with employer-provided insurance and those with Medicaid plans show dramatically different risks [adjusted odds ratio (AOR): 0.761 vs 1.41]. Low-income groups demonstrate higher risk (household income less than $25k, AOR: 1.3-1.57) than high-income groups ($100-$200k, AOR: 0.597-0.867). Other high-risk factors include impaired mobility (AOR: 1.32), unemployment (AOR: 1.32), renters (AOR: 1.41), other non-house-owners (AOR: 1.7), and house instability (AOR: 1.25). Education was negatively associated with acidosis risk.

**Discussion:**

Our work provides real-world evidence of the comprehensive health disparities due to socioeconomic and behavioral contributors in a cohort enriched in minority groups or underrepresented populations.

**Conclusions:**

SDoHs are strongly associated with systematic health disparities in the risk of severe metabolic acidosis. Types of health insurance, household income levels, housing status and stability, employment status, educational level, and mobility disability play significant roles after being adjusted for demographic features and clinical conditions. Comprehensive solutions are needed to improve equity in healthcare and reduce the risk of severe acidosis.

## Introduction

Metabolic acidosis is a clinical condition in which the body generates or retains an excess of hydrogen ions or is deficient in bicarbonate. This condition is often related to disturbed lactate metabolism, ketone accumulation, ingestion of compounds, renal tubular acidosis, stool bicarbonate losses, or retained organic anions in organ failure.^1–3^ Severe metabolic acidosis during hospitalization or emergency care portends high mortality. For example, acidosis with blood lactate concentrations >5 mmol/L holds an estimated mortality rate of 80% in critically ill patients.^2^ Risk factors for severe metabolic acidosis include hypoxic cases (type A lactic acidosis) such as shock, ischemia, seizures, critical illness,^2^^,^^4^ and sepsis,^5^ and non-hypoxic cases (type B lactic acidosis) related to liver disease,^6^^,^^7^ drugs, and toxins such as metformin,^8^ blood cancers,^2^ and genetic errors that cause mitochondrial myopathy or pyruvate dehydrogenase deficiency.^2^ Other causes of acidosis include diabetic ketoacidosis,^9^ decreased glomerular filtration rate,^10–12^ severe renal tubular acidosis related to hyporeninemia, and hyperkalemia. The acidosis prevalence is estimated to be 37% in individuals with stage 4 chronic kidney disease (CKD),^4^ 13%-80% in diabetic patients,^13^ and over 25% in sepsis cases (MIMIC-IV dataset,^14^ in-house analysis). Social determinants of health (SDoHs), such as health insurance type, disabilities, employment status, income level, housing status, and education level can all affect access to care, which can reduce monitoring and increase the likelihood of hospitalization. For example, CKD disproportionally affects those with lower socioeconomic status and education levels.^15^ However, health disparities and the role of SDoH in severe acidosis have not been comprehensively examined.

The genetic traits associated with severe acidosis are also understudied. Transporters involved in the movement of nutrients and the pharmacokinetics of drugs (such as metformin) in the kidney, liver, and intestine may be relevant.^8^ Some candidate transporters include organic ion transporters OCT1,^16^^,^^17^ OCT2,^18^ and OCTN1^19^^,^^20^, and multidrug and toxin extrusion transporters MATE1^19^^,^^21^ and MATE2K.^22^^,^^23^ The independent roles of these genetic traits in the context of demographic and clinical conditions have not been fully explored in larger datasets.

We hypothesized that the NIH-funded *All of Us* Research Program^24^ would prove to be an important tool to understand the SDoHs that contribute to the risk of severe acidosis. The *All of Us* data cover longitudinal electronic health records (EHRs) that delineate the trends of clinical conditions, genomics data for genetic traits, and survey data for SDoH and lifestyles. More importantly, the *All of Us* cohort is recruited from minority groups and historically underrepresented populations in biomedical research. With its current version containing over 400 000 participants from across the United States, the *All of Us* research data allow for holistic profiling of the roles of SDoHs in the risk of severe acidosis.

Our work, for the first time, utilizes propensity score matching approaches on *All of Us* multi-domain EHR, genomics, and survey data to systematically examine major SDoHs and candidate genetic traits in the context of known demographic and clinical risk factors. Our discoveries cast new light on how to diminish health disparities in severe acidosis and manage the risk for the various causes of metabolic acidosis.

## Methods

**Figure 1.**
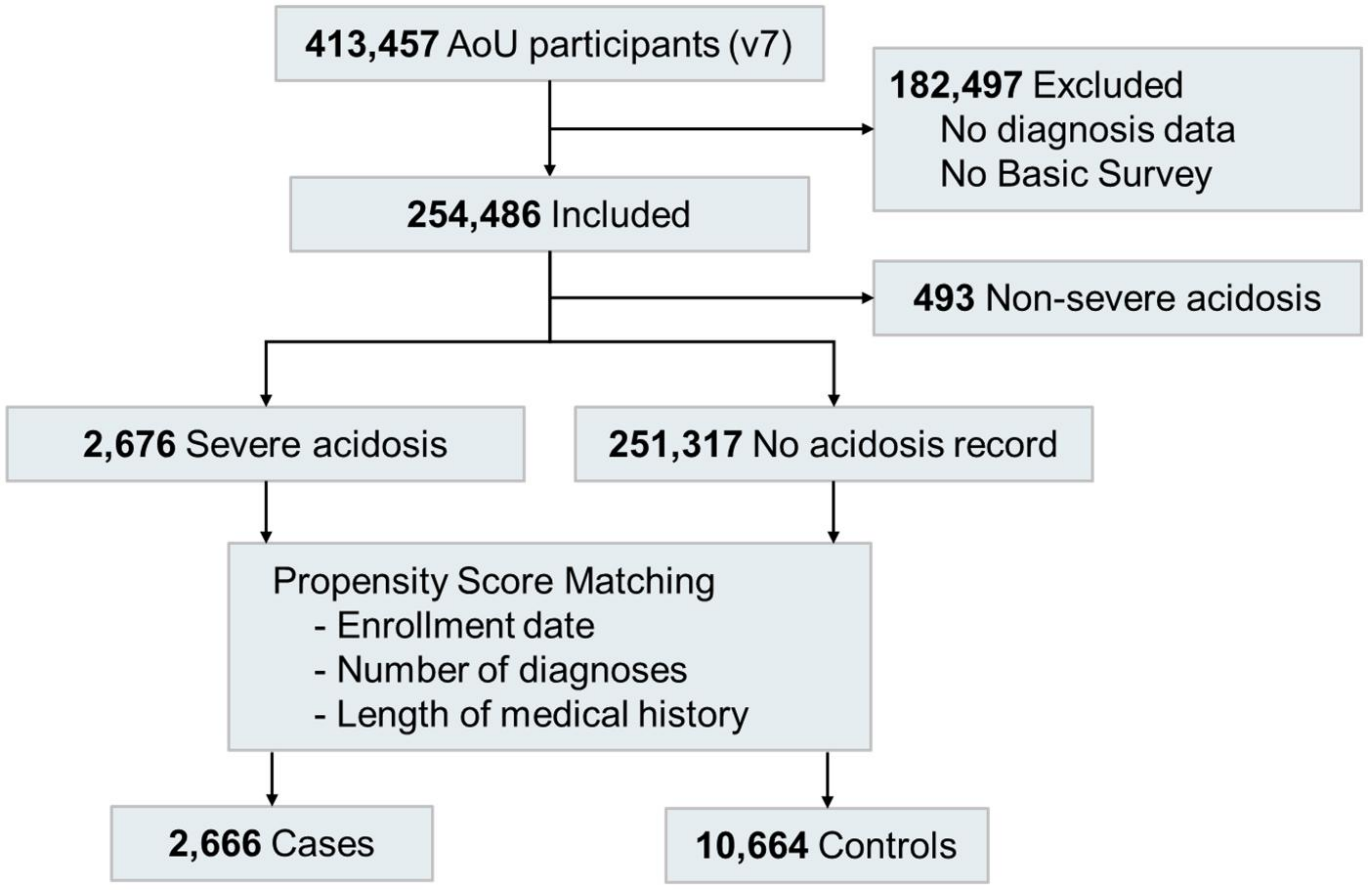
Study participant flowchart.

This study uses the *All of Us* Research Program^24^ data following the Strengthening the Reporting of Observational Studies in Epidemiology (STROBE) reporting guideline for observational studies.^25^ A propensity score matching approach and conditional logistic regression are used to delineate the role of SDoHs in health disparities. The study participant flowchart is shown in Figure 1.

### Study design and participants

A retrospective case-control design is used to comprehensively examine the roles of SDoHs and candidate genotypes for the risk of developing severe, acute acidosis. The latest multi-domain *All of Us* Controlled Tier Data are used in this study. Specifically, we use the Curated Data Repository version 7 C2022Q4R9, established in April 2023, with a cutoff date of July 1, 2022. The conditions, drug exposures, labs and measurements, and visits in the EHR domains, the Basics in the Survey domain, and the whole genome sequencing (WGS) data in the Genomics domain are used. Observational Medical Outcomes Partnership (OMOP) Common Data Model (CDM) version 5.3.1 is utilized in data preparation.

Individuals who had severe, acute acidosis incidences, as well as both diagnosis and Basics Survey data, are selected as cases. Those who had no acidosis records are used as candidates for controls. A case-control propensity matching with a 1:4 ratio is conducted utilizing *All of Us* enrollment date, number of diagnoses, and length of medical history.

### Outcome

Severe and acute acidosis is the primary outcome of this study. The International Classification of Diseases version 9 (ICD-9) and version 10 (ICD-10) codes in the Conditions domain and the OMOP Visit codes (Tables S1 and S2), as well as the phenotyping algorithm (Figure S1), are provided in Supplementary Material S1.

### Exposure and covariates

Demographic features such as sex at birth, race, ethnicity, and age at acidosis, drug exposure for metformin use (Table S3), clinical conditions such as Charlson comorbidities (Table S4),^26^ SDoHs such as health insurance, disability, employment, housing, housing stability, and education level (Table S5), as well as risk genotypes of genes MATE1/SLC47A1 (rs2289669, rs8065082, and rs8065082), MATE2K (rs12943590), OCTN1 (rs272893), OCT1 (rs12208357, rs72552763, and rs622342), and OCT2 (rs316019) (Table S6) are extracted. Code sets are summarized in Supplementary Material S1 (Table S1 and Figure S1: code sets and algorithm for severe/acute acidosis; Table S5: code set for metformin; Table S4: code sets for Charlson comorbidities; Table S5: code sets for SDoH; and Table S6: acidosis-related genotypes). The concordance of sex and gender identity is summarized in Table S7. Missing data processing is discussed in the Supplementary Method.

### Statistical analysis

The case-control matched data are analyzed using the conditional logistic regression model. A base model is created using the following covariates: sex at birth, race, ethnicity, age, metformin use, and Charlson comorbidities. Derived from the base model, individual analyses are performed for each SDoH factor and acidosis-related genotype.

## Results

### Participant characteristics

Our cohort comprises 2666 patients in the case group and 10 664 in the matched control group (Figure 1). The following groups of participants were excluded from the cohort: those with no EHR data availability, those who did not complete *All of Us* Basic Survey, and those who did not experience severe acidosis. The similar distributions of longitudinal EHR data availability of the case and the control groups (Tables S8 and S9, Figure S2, and Supplementary Method) suggest good control of confounding effects due to the completeness of the EHR data, the enrollment date, and the length of medical history. Participants’ demographic characteristics and clinical conditions (Table 1) and SDoH statuses (Table 2) are summarized for both the case and control groups.

**Table 1.** The characteristics of demographic features and clinical conditions.

Characteristic	Control group (*n* = 10 664)	Case group (*n* = 2666)
**Sex at birth (*n*, %)**		
Male	3455, 32.4%	1221, 45.8%
Female	6936, 65.0%	1390, 52.1%
Other	273, 2.6%	55, 2.1%
**Race (*n*, %)**		
White	6384, 59.9%	1065, 39.9%
Black	2007, 18.8%	842, 31.6%
Asian	204, 1.9%	34, 1.3%
Other	2069, 19.4%	725, 27.2%
**Ethnicity (*n*, %)**		
Not Hispanic	8671, 81.3%	1947, 73.0%
Hispanic	1547, 14.5%	607, 22.8%
Other	446, 4.2%	112, 4.2%
**Age**		
Median, IQR (years)	51, 38-61	51, 42-60
<45 years (*n*, %)	3932, 36.9%	829, 31.1%
46-55 years (*n*, %)	2389, 22.4%	766, 28.7%
56-65 years (*n*, %)	2367, 22.2%	693, 26.0%
65+ (*n*, %)	1976, 18.5%	378, 14.2%
**Metformin use (*n*, %)**	337, 3.2%	577, 21.6%
**Comorbidities (*n*, %)**		
Liver disease (mild)	2701, 25.3%	1037, 38.9%
Liver disease (moderate-severe)	296, 2.8%	244, 9.2%
Renal disease (mild-moderate)	1704, 16.0%	973, 36.5%
Renal disease (severe)	835, 7.8%	686, 25.7%
Diabetes (without complications)	1682, 15.8%	540, 20.3%
Diabetes (with complications)	2093, 19.6%	2066, 77.5%
Myocardial infarction	1369, 12.8%	910, 34.1%
Congestive heart failure	2134, 20.0%	1346, 50.5%
Peripheral vascular disease	2382, 22.3%	1100, 41.3%
Cerebrovascular disease	2155, 20.2%	789, 29.6%
Dementia	357, 3.3%	174, 6.5%
Chronic pulmonary disease	5368, 50.3%	1689, 63.4%
Rheumatic disease	1438, 13.5%	374, 14.0%
Peptic ulcer disease	995, 9.3%	417, 15.6%
Hemiplegia/paraplegia	513, 4.8%	268, 10.1%
HIV	98, 0.9%	30, 1.1%
AIDS	172, 1.6%	88, 3.3%
Malignancy	2286, 21.4%	588, 22.1%
Metastatic solid tumor	586, 5.5%	208, 7.8%

**Table 2.** The characteristics of social determinants of health.

Characteristic	Control group (*n* = 10 664)	Case group (*n* = 2666)
**Health insurance (*n*, %)**		
Employer	2967, 27.8%	368, 13.8%
Medicare	2874, 27.0%	892, 33.5%
Medicaid	1588, 14.9%	689, 25.8%
**Disability (*n*, %)**		
No disabilities	1364, 12.8%	270, 10.1%
Any disability	856, 8.0%	427, 16.0%
Vision	164, 1.5%	100, 3.8%
Hearing	235, 2.2%	112, 4.2%
Mobility	525, 4.9%	331, 12.4%
Self-care	154, 1.4%	95, 3.6%
Independent living	230, 2.2%	131, 4.9%
Cognition	322, 3.0%	138, 5.1%
**Employment (*n*, %)**		
Employed	3092, 29.0%	402, 15.1%
Student	173, 1.6%	21, 0.8%
Unemployed	2730, 25.6%	1199, 45.0%
Others	4307, 40.4%	966, 36.2%
**Income (*n*, %)**		
Less than 10k	1349, 12.7%	523, 19.6%
10-25k	1488, 14.0%	568, 21.3%
25-35k	793, 7.4%	194, 7.3%
35-100k	2737, 25.7%	452, 17.0%
100-150k	1033, 9.7%	114, 4.3%
150-200k	454, 4.3%	28, 1.1%
More than 200k	646, 6.1%	40, 1.5%
**Housing (*n*, %)**		
Owner	5262, 49.3%	825, 30.9%
Rent	4142, 38.8%	1473, 55.3%
Others	788, 7.4%	250, 9.4%
**Housing stability (*n*, %)**		
Stable	8840, 83.0%	2043, 76.6%
Unstable	1578, 14.8%	556, 20.1%
**Education (*n*, %)**		
Never attended	<20, <0.19%	<20, <0.75%
Below GED	910, 8.5%	510, 19.1%
GED or college	4845, 45.4%	1430, 53.6%
College graduate or advanced	4552, 42.7%	605, 22.7%

In the case group, we observe higher percentages of males (45.8% vs 32.4%), African American participants (31.6% vs 18.8%), and Hispanic Americans (22.8% vs 14.5%) compared to the control group, suggesting noticeable health disparities of acidosis risk in these demographic groups. Metformin use is 6-fold higher in the case group (21.6%) than in the control group (3.2%), representing the importance of metformin-associated lactic acidosis. Liver diseases, renal diseases, diabetes, cardiovascular diseases, peptic ulcer disease, hemiplegia and paraplegia, and AIDS are more prevalent in the case group than in the control group.

SDoHs demonstrate strong influences on severe acidosis risks (Table 2). For example, compared with the control group, the case group demonstrates lower percentages of employment (15.1% vs 29.0%) and higher percentages of housing instability (20.1% vs 14.8%). Overall, education and income levels are lower in the case group.

### The roles of demographics, metformin exposure, and comorbidities

**Figure 2.**
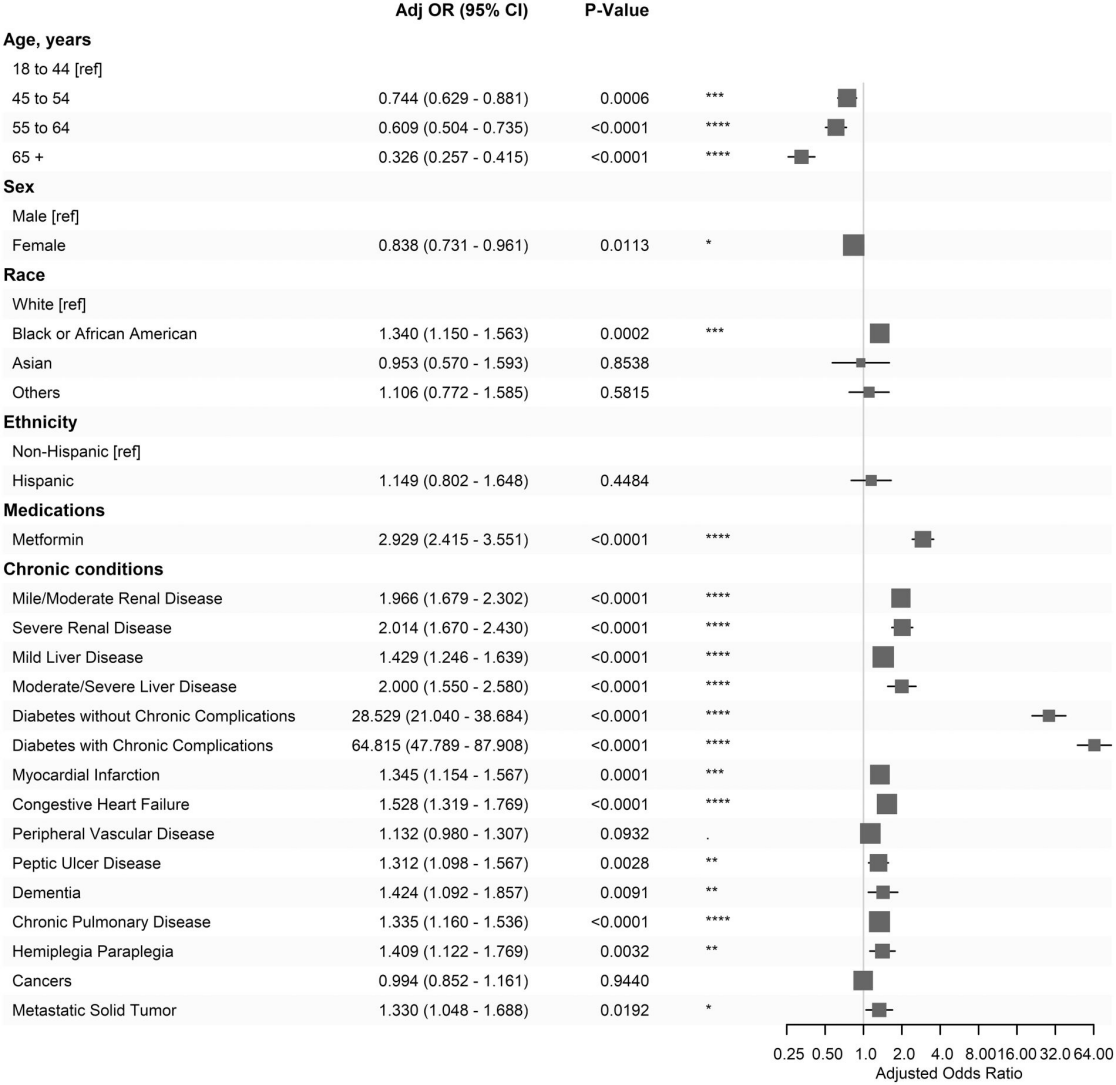
The base model demonstrates the associations of demographics, metformin exposure, and comorbidities with the severe acidosis risk.

A comprehensive analysis of the roles of demographics, metformin use, and comorbidities in the base model, which is summarized in Figure 2, provides new insights into the observed differential percentages. Health disparities in African American participants (adjusted odds ratio or AOR = 1.34) remain significant. Interestingly, after adjusting for comorbidities, the acidosis risk in middle-aged and senior groups is lower than the reference age group of 18-44 years, with age groups 45-54 (AOR = 0.744), 55-64 (AOR = 0.609), and 65+ (AOR = 0.326). Metformin use (AOR = 2.93) remains a significant acidosis risk factor. Renal diseases (AOR = 1.97-2.01), liver disease (AOR = 1.25-2.58), diabetes (AOR = 28.5-64.8), dementia (AOR = 1.42), chronic pulmonary disease (AOR = 1.33), myocardial infarction (AOR = 1.34), congestive heart failure (AOR = 1.53), hemiplegia paraplegia (AOR = 1.41), and metastatic solid tumor (AOR = 1.33) are all associated with increased acidosis risk. Interestingly, patients with non-metastatic cancers or peripheral vascular diseases are not at elevated acidosis risk (Figure S3).

### The contributions of individual SDoH factors adjusted for demographics and clinical conditions

**Figure 3.**
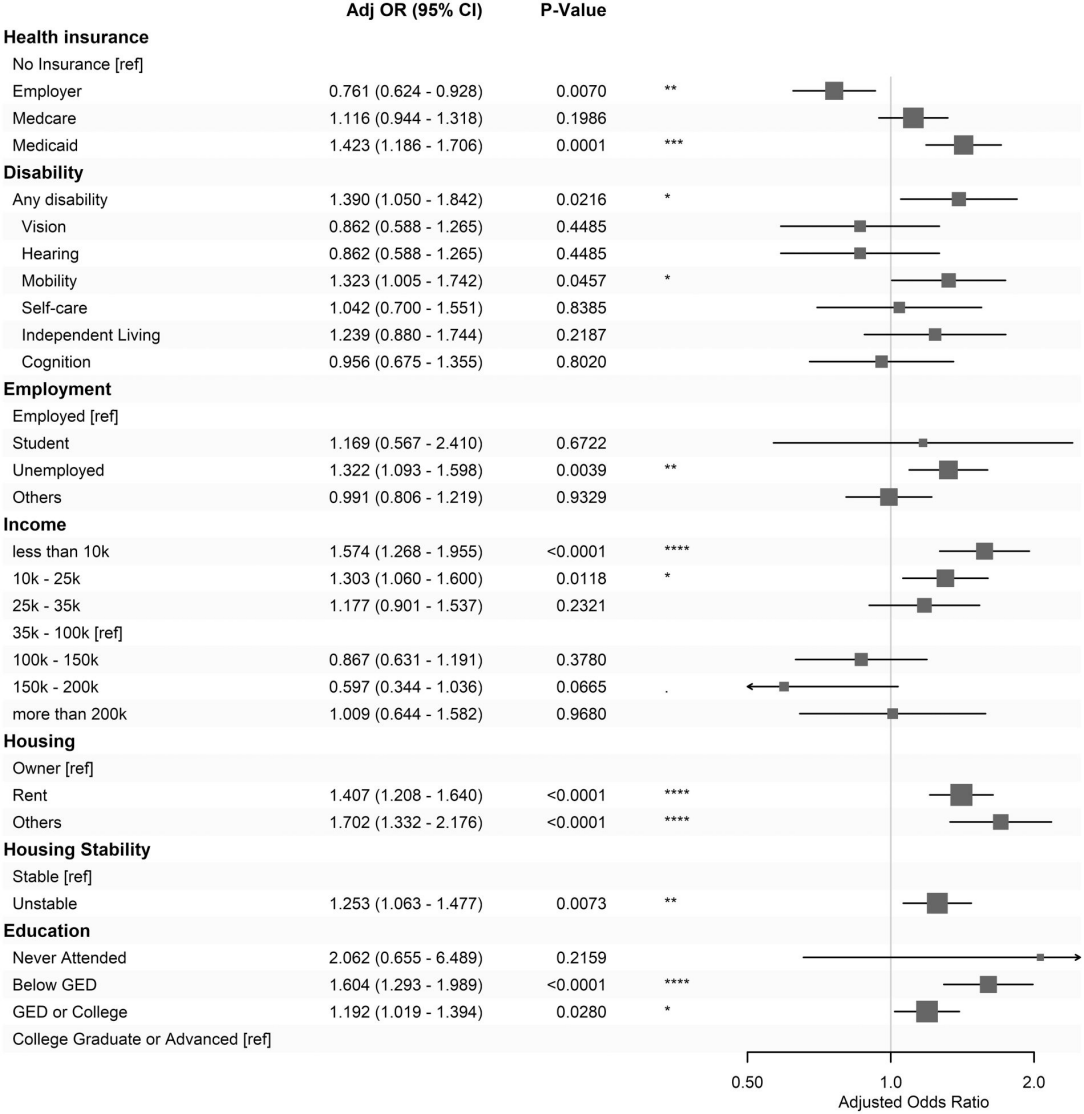
The social determinants of health (SDoH) models for the severe acidosis risk, after adjusting for demographics, metformin exposure, and comorbidities.

Each SDoH factor is evaluated within the base model, and the overall results of these 12 models are summarized in Figure 3. All analyzed SDoHs significantly contribute to the health disparity in the risk of severe acidosis. Compared to those without insurance, those with employer-provided insurance have a decreased risk of acidosis (AOR = 0.761), while those with Medicaid are at higher risk (AOR = 1.42). Participants who have impaired mobility demonstrate an increased risk of acidosis (AOR = 1.32). Unemployment is a risk factor (AOR = 1.32). Low-income groups are more likely to develop severe acidosis (household income of $10-$25k: AOR = 1.3; less than $10k: AOR = 1.57). Renters (AOR: 1.41) and other non-owners (AOR: 1.7) are risk populations. Housing instability is associated with a higher risk (AOR = 1.25). Education levels are negatively associated with acidosis risk. Compared to those who have college or advanced degrees, those who have education levels of or below GED (General Education Development) exhibit an increased risk (AOR = 1.6).

### Associations of candidate genotypes adjusted for demographics and clinical conditions

**Figure 4.**
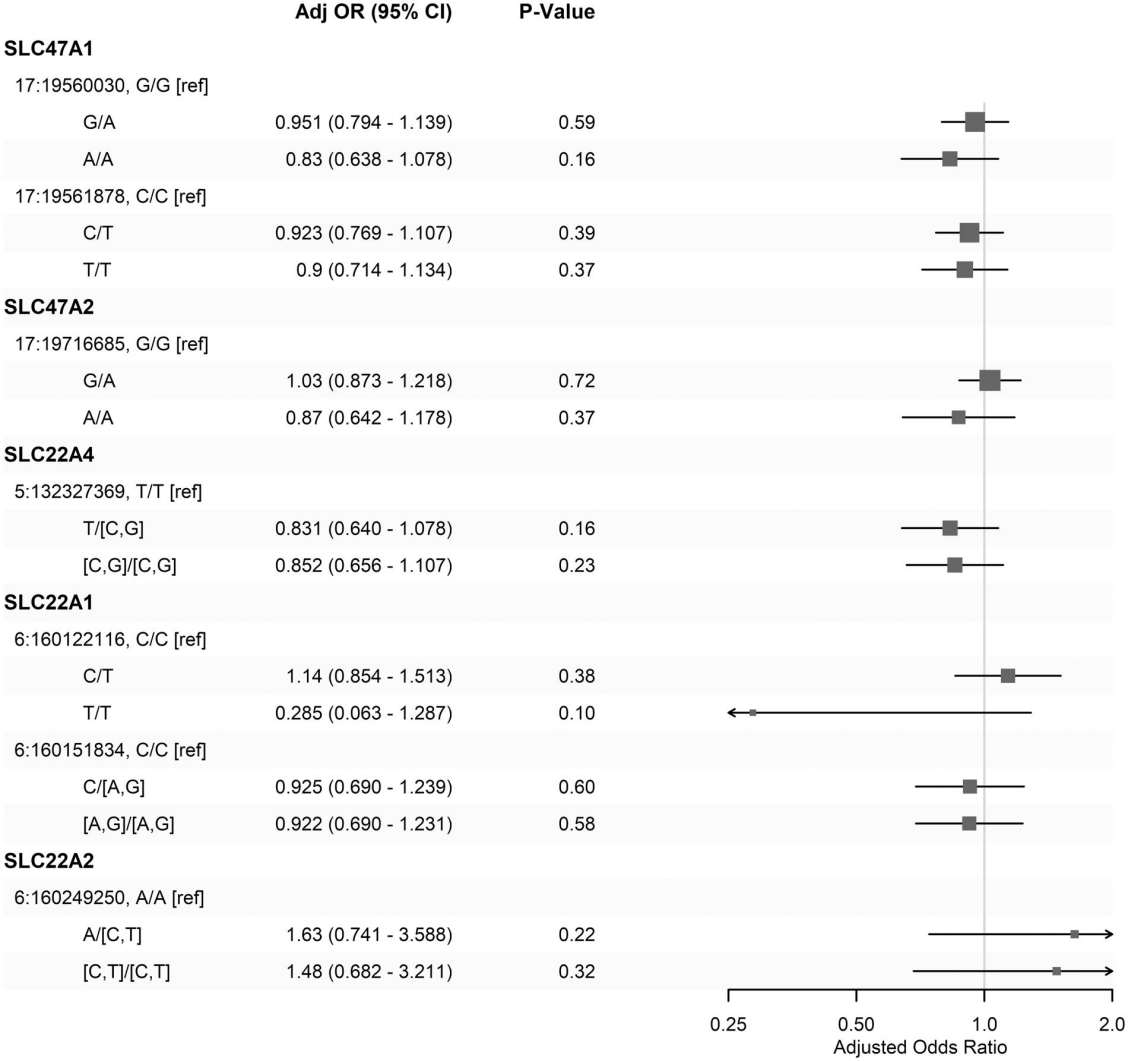
The associations of genetic traits for the severe acidosis risk, after adjusting for demographics, metformin exposure, and comorbidities.

The selected known risk genotypes of transporters responsible for the transportation of organic anions and cations that potentially affect the pharmacokinetics of metformin do not show statistically significant associations with the risk of severe acidosis (Figure 4). Among them, the T/T homozygous variant at 17:160122116 of gene SLC22A1 demonstrates a strong but widely diverse low risk (AOR: 0.285, 95% CI, 0.0633-1.290). Similarly, the A>C or A>T variants at 6:160249250 of SLC47A2 are associated with higher but also diverse risks (AOR: 1.48 and 1.63, 95% CI, 0.741-3.590 and 0.682, 3.210, respectively). Further investigations in larger cohorts and specific populations are necessary to understand the role of these genotypes in severe acidosis.

## Discussion

Severe acidosis is a life-threatening emergency and public health indicator of a wide range of underlying factors, including limited access to healthcare,^27^ unhealthy lifestyles, poor health literacy,^28^ poor management of chronic conditions, various pathological causes, and genetic factors. Our epidemiological profiling of the roles of SDoH in the context of demographic and clinical conditions in the underrepresented population provides the landscape of health disparities and paves the way for future investigations in specific acidosis types and clinical conditions.

### Return of value to communities

Our work, for the first time, revealed the community health disparities of severe acidosis, a rare but fatal clinical emergency. The comprehensive modeling incorporated SDoH, EHR, and genomic data. Our work provides real-world evidence to elucidate the socioeconomic and behavioral contributors to health disparities because essential healthcare is not accessible.^27^ The limited access to necessary healthcare can be mitigated by precision public health policies to reduce the barriers to accessing treatments and services for high-risk populations.^29^ The lack of timely utilization of available and affordable healthcare services for managing chronic conditions is related to behavioral problems and health literacy, and it can be improved through personal behavioral interventions and health education. For patients who have access to healthcare and with similar clinical conditions, the overall EHR frequency of a patient is more associated with lifestyles and personal health literacy, while the difference in the types of healthcare services is associated with available and affordable choices in chronic disease management and treatment. Our propensity score matching design elucidates the barriers to accessing necessary healthcare by disentangling and controlling the confounding effects related to patients’ willingness to use healthcare services. Thus, our results illustrate the systematic health barriers underlying the observed health disparities in the risk of severe acidosis, which cannot be eliminated by health interventions and education but need changes in public health policies.

The significant associations between insurance types and severe acidosis types suggested that, for patients who have a similar willingness and frequency to use healthcare, employer-provided insurance covers crucial services for avoiding severe acidosis, which are not available or affordable for Medicaid users.^30^ The strong risk patterns in household incomes suggest that financial barriers limit healthcare choices for those who have a similar frequency of using healthcare. The healthcare choices of patients with impaired mobility are limited by the adjacency to healthcare services, which may play a role in their significantly increased severe acidosis risk. Taken together, the lack of choices of necessary healthcare plays a crucial and independent role in the observed health disparities. Further investigation into the specific healthcare services will provide evidence to improve current health policies.

The initial exploration of selected known risk genotypes of transporters suggests the potentially significant roles of SLC22A1 and SLC47A2 in acidosis, but further investigations into the risk-specific subpopulations are necessary. Thorough and sophisticated genome-wide association studies are promising for identifying risk genotypic markers and building acidosis-specific polygenic risk scores.

### Strengths

The major strength of this work is the first comprehensive analysis of SDoH in health disparities, which leverages the unique *All of Us* multimodal data of the population of minority groups and underrepresented populations. The propensity score matching design allows for the inference of systematic health disparities that demand changes in public health policies. This is also the first work profiling the associations of the 6 categories of disabilities with severe acidosis. This work provides novel knowledge and real-world evidence on the complexity of health disparities and enables future studies into specific types of acidosis, health interventions, and genotypes.

### Limitations

We observed common limitations of using EHR data in observational studies, such as data incompleteness, changes in clinical guidelines and practices with time, heterogeneous data granularity, and reporting biases of events. We carefully designed the study to reduce artifacts and biases. For example, the metformin records were likely to be incomplete, indicated by the low drug use in the case group (21.6% participants), considering most participants in this group (97.8%) had evidence of diabetes mellitus. The guidelines, printed labeling approved by the Food and Drug Administration (FDA), and scientific evidence of contraindication of metformin use for patients with renal deficiency have changed in recent years, from the estimated glomerular filtration rate (eGFR) below 60 mL/min/1.73 m^2^ in the year 1994 to below 30 mL/min/1.73 m^2^ in the year 2014.^31^ We used propensity score matching to control unobserved confounding effects due to EHR data availability and completeness, as well as clinical practice changes. Diagnosis, lab measurements, drugs, and procedures can potentially provide information on acidosis subtypes such as lactic, hyperchloremic, renal tubular, and diabetic ketoacidosis. However, the granularity of EHR data in these domains was heterogeneous across providers. Also, currently, there is no reliable algorithm to distinguish community-acquired acidosis from ICU- or hospital-acquired respiratory acidosis in EHR records. Since this study focuses on health disparities, we decided not to phenotype acidosis subtypes to avoid introducing granularity-induced biases. To reduce the reporting bias of acidosis incidences, we focused on severe and acute acidosis, defined as emergency visits, hospitalization, or urgent care incidences. Furthermore, the *All of Us* Research Program focuses on health disparities among underrepresented and underserved populations. Thus, caution should be paid when generalizing the discovered knowledge of acidosis health disparities to the general population.

## Conclusions

SDoH are strongly associated with systematic health disparities in the risk of severe acidosis. Types of health insurance, household income levels, housing status and stabilities, employment status, educational levels, and mobility disabilities play significant roles after being adjusted for demographic features and clinical conditions.

## Supplementary Material

ocae256_Supplementary_Data

## Data Availability

All data and codes are available at *All of Us* Research Workbench under the workspace “Acidosis Risk Health Disparities” for users who are authorized to access *All of Us* Controlled Tier Data v7. Codes for reproducing the analysis are also available at https://github.com/Su-informatics-lab/aou_acidosis.
