# Supplementary material for: Health disparities in the risk of severe acidosis: real-world evidence from the All of Us cohort
Source: J Am Med Inform Assoc. 2024 Oct 14;31(12):2932–9. doi: 10.1093/jamia/ocae256 (PMC11631078; doi:10.1093/jamia/ocae256)

**Supplementary Materials**

Gatz, Su, et al, Health Disparities in the Risk of Severe Acidosis: Real-world Evidence from the All of Us Cohort

**Contents**

[eTable 1. Code sets for severe/acute acidosis. 2](#_Toc1926054418)

[eTable 2. Distributions of Visit Types of Acidosis Encounters 3](#_Toc49695308)

[eFigure 1. Algorithm for phenotyping severe/acute acidosis incidences 4](#_Toc985991115)

[eTable 3: Code set for metformin. 5](#_Toc1203061516)

[eTable 4: Code sets for Charlson comorbidities. 6](#_Toc1943321663)

[eTable 5: Code sets for SDoH. 26](#_Toc1795252465)

[eTable 6: Acidosis-related genotypes. 30](#_Toc518369466)

[eTable 7: Sex Assigned at Birth and Gender Identity Concordance. 31](#_Toc51399374)

[eMethod 32](#_Toc2095657833)

[eTable 8: Pre-matching sample balance table 33](#_Toc403037719)

[eTable 9: Post-matching sample balance table 34](#_Toc1087707321)

[eFigure 2. Longitudinal availability of diagnosis data in the case and the control groups 35](#_Toc1540350681)

[eFigure 3. Adjusted odds ratios of the base model. 36](#_Toc1422850057)

# eTable 1. Code sets for severe/acute acidosis.

| **Phenotype** | **Code Type** | **Code** |
| --- | --- | --- |
| Acidosis | ICD9 | 276.2 |
|  | ICD10 | E87.2 |
| Severe/acute visit | OMOP | 9203: Emergency Room Visit  262: Emergency Room and Inpatient Visit  9201: Inpatient Visit  8717: Inpatient Hospital  8782: Urgent Care Facility |

# eTable 2. Distributions of Visit Types of Acidosis Encounters

Severe or acute visit types are annotated in red.

| **STANDARD_CONCEPT_NAME** | **# of Records** | **%** |
| --- | --- | --- |
| Outpatient Visit | 924,206 | 67.70 |
| Office Visit | 148,640 | 10.89 |
| **Inpatient Visit** | **104,522** | **7.66** |
| Laboratory Visit | 56,712 | 4.15 |
| **Emergency Room Visit** | **56,357** | **4.13** |
| Telehealth | 32,770 | 2.40 |
| Ambulatory Rehabilitation Visit | 8,669 | 0.64 |
| **Emergency Room and Inpatient Visit** | **7,984** | **0.58** |
| Non-hospital institution Visit | 6,232 | 0.46 |
| Pharmacy visit | 5,782 | 0.42 |
| Home Visit | 4,984 | 0.37 |
| Ambulatory Radiology Clinic / Center | 2,389 | 0.17 |
| **Inpatient Hospital** | **2,090** | **0.15** |
| Outpatient Hospital | 1,141 | 0.084 |
| Ambulatory Surgical Center | 841 | 0.062 |
| Ambulatory Infusion Therapy Clinic / Center | 401 | 0.029 |
| Observation Room | 326 | 0.024 |
| Hospital | 324 | 0.024 |
| Unknown Value (but present in data) | 132 | 0.010 |
| Case Management Visit | 112 | 0.008 |
| Ambulatory Oncology Clinic / Center | 98 | 0.007 |
| Emergency Room - Hospital | 90 | 0.007 |
| Ambulatory Mammography Clinic / Center | 76 | 0.006 |
| Ambulatory Magnetic Resonance Imaging (MRI) Cl... | 64 | 0.005 |
| Ambulatory Endoscopy Clinic / Center | 44 | 0.003 |
| Radiation Therapy Center | 40 | 0.003 |
| Rehabilitation Hospital | 31 | 0.002 |
| Nursing Facility | 24 | 0.002 |
| Health Examination | <20 | <0.002 |
| **Urgent Care Facility** | **<20** | **<0.002** |
| Mass Immunization Center | <20 | <0.002 |
| Ambulatory Research Clinic / Center | <20 | <0.002 |
| Ambulatory Ophthalmologic Surgery Clinic / Center | <20 | <0.002 |
| Behavioral Disturbances Assisted Living Facility | <20 | <0.002 |
| Home Health Agency | <20 | <0.002 |
| Ambulatory Clinic / Center | <20 | <0.002 |

# eFigure 1. Algorithm for phenotyping severe/acute acidosis incidences


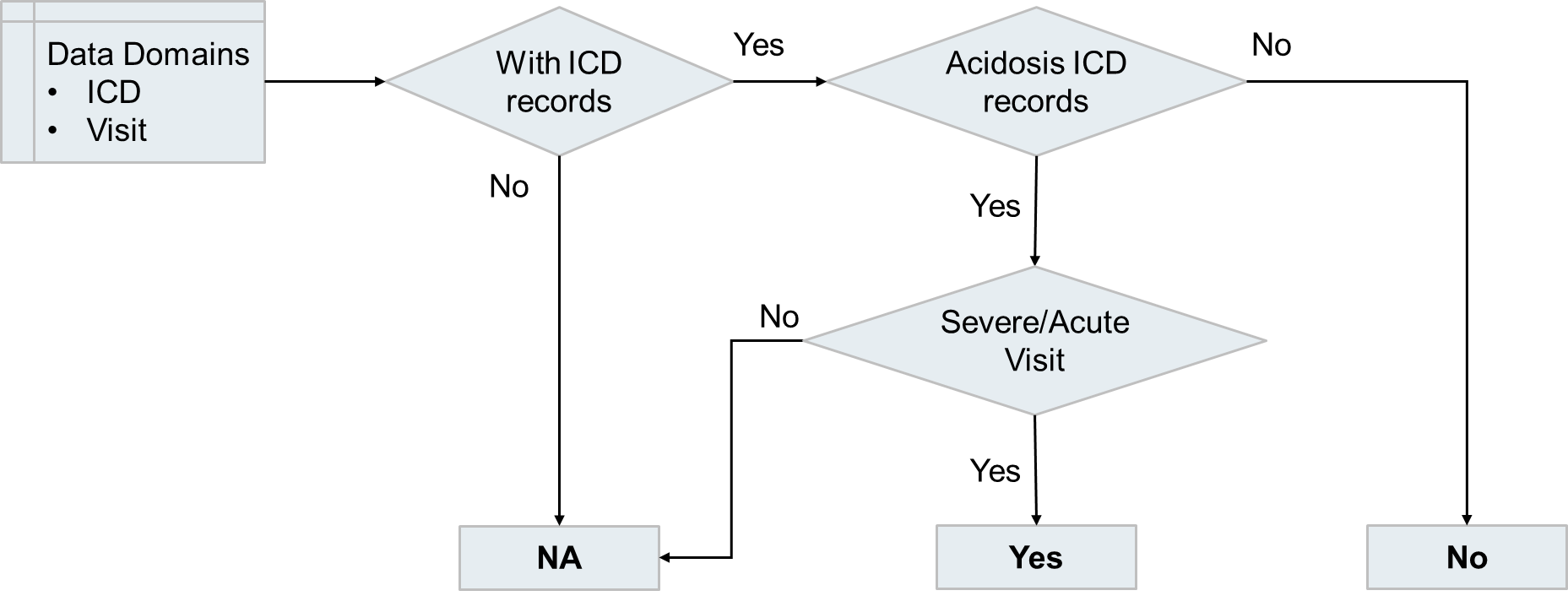


# eTable 3: Code set for metformin.

| Medication | Code Type | Code |
| --- | --- | --- |
| Metformin | OMOP | 1503297 |

# eTable 4: Code sets for Charlson comorbidities.

Reproduced from *Glasheen et al, Charlson Comorbidity Index: ICD-9 Update and ICD-10 Translation. Am Health Drug Benefits. 2019*, with the permission of the author (Glasheen) and the journal *American Health & Drug Benefits*.

Hi Allison – could you reproduce the tables SI-3a through SI-3s in “ahdb-12-188-s1.doc” please? Please remove the column “ICD-9 Diagnosis Code (Deyo et al., 1992)” and only keep the columns “ICD-9 Diagnosis Code (CDMF CCI)” and “ICD-10 Diagnosis Code (CDMF CCI)”.

*eTable 4a. Deyo and CDMF CCI Coding Schemes: Myocardial Infarction*

| **ICD-9 Diagnosis Code** | | **ICD-10 Diagnosis Code** | |
| --- | --- | --- | --- |
| 410.x  412.x | Acute Myocardial Infarction  Old Myocardial Infarction | I21.x  I22.x  I25.2 | STEMI and NSTEMI  Subsequent STEMI and NSTEMI  Old Myocardial Infarction |

*eTable 4b. Deyo and CDMF CCI Coding Schemes: Congestive Heart Failure*

| **ICD-9 Diagnosis Code** | | **ICD-10 Diagnosis Code** | |
| --- | --- | --- | --- |
| 398.91  402.01    402.11    402.91    404.01      404.03  404.11      404.13  404.91        404.93      425.4  425.5  425.6  425.7  425.8  425.9  428.x | Rheumatic heart failure (congestive)  Malignant hypertensive heart disease with heart failure  Benign hypertensive heart disease with heart failure  Unspecified hypertensive disease with heart failure  Hypertensive heart and chronic kidney disease, malignant, with heart failure and with chronic kidney disease stage I through stage IV, or unspecified  Hypertensive heart and chronic kidney disease, malignant, with heart failure and with chronic kidney disease stage V or end stage renal disease  Hypertensive heart and chronic kidney disease, benign, with heart failure and with chronic kidney disease stage I through stage IV, or unspecified  Hypertensive heart and chronic kidney disease, benign, with heart failure and chronic kidney disease stage V or end stage renal disease  Hypertensive heart and chronic kidney disease, unspecified, with heart failure and with chronic kidney disease stage I through stage IV, or unspecified  Hypertensive heart and chronic kidney disease, unspecified, with heart failure and chronic kidney disease stage V or end stage renal disease  Other primary cardiomyopathies  Alcoholic cardiomyopathy  Cardiomyopathy in Chagas disease  Nutritional and metabolic cardiomyopathy  Cardiomyopathy in other disease classified elsewhere  Secondary cardiomyopathy, unspecified  Heart failure | I11.0    I13.0          I13.2        I25.5  I42.0  I42.5  I42.6  I42.7    I42.8  I42.9  I43.x    I50.x  P29.0 | Hypertensive heart disease with heart failure  Hypertensive heart and chronic kidney disease with heart failure and stage 1 through stage 4 chronic kidney disease, or unspecified chronic kidney disease  Hypertensive heart and chronic kidney disease with heart failure and with stage 5 chronic kidney disease, or end stage renal disease  Ischemic cardiomyopathy  Cardiomyopathy  Other restrictive cardiomyopathy  Alcoholic cardiomyopathy  Cardiomyopathy due to drug and external agent  Other cardiomyopathies  Cardiomyopathy, unspecified  Cardiomyopathy in diseases classified elsewhere  Heart failure  Neonatal cardiac failure |

*eTable 4c. Deyo and CDMF CCI Coding Schemes: Peripheral Vascular Disease*

| **ICD-9 Diagnosis Code** | | **ICD-10 Diagnosis Code** | |
| --- | --- | --- | --- |
| 093.0    437.3  440.x  441.x  443.1  443.2x    443.8x    443.9  447.1  557.1  557.9  V43.4 | Aneurysm of aorta, specified as syphilitic  Cerebral aneurysm, non-ruptured  Atherosclerosis  Aortic aneurysm and dissection  Thromboangiitis obliterans [Buerger's disease]  Other arterial dissection  Other specified peripheral vascular diseases  Peripheral vascular disease, unspecified  Stricture of artery  Chronic vascular insufficiency of intestine  Unspecified vascular insufficiency of intestine  Blood vessels replaced by other means | I70.x  I71.x  I73.1    I73.8    I73.9    I77.1  I79.0    I79.1    I79.8      K55.1  K55.8  K55.9    Z95.8    Z95.9 | Atherosclerosis  Aortic aneurysm and dissection  Thromboangiitis obliterans [Buerger's disease]  Other specified peripheral vascular diseases  Peripheral vascular disease, unspecified  Stricture of artery  Aneurysm of aorta in diseases classified elsewhere  Aortitis in diseases classified elsewhere  Other disorders of arteries, arterioles, and capillaries in diseases classified elsewhere  Chronic vascular disorders of intestine  Other vascular disorders of intestine  Vascular disorder of intestine, unspecified  Presence of other cardiac and vascular implants and grafts  Presence of cardiac and vascular implant and graft, unspecified |

*eTable 4d. Deyo and CDMF CCI Coding Schemes: Cerebrovascular Disease*

| **ICD-9 Diagnosis Code** | | **ICD-10 Diagnosis Code** | |
| --- | --- | --- | --- |
| 362.34  430.x  431.x  432.x    433.x    434.x  435.x  436.x    437.x    438.x | Transient retinal arterial occlusion  Subarachnoid hemorrhage  Intracerebral hemorrhage  Other and unspecified intracranial hemorrhage  Occlusion and stenosis of precerebral arteries  Occlusion of cerebral arteries  Transient cerebral ischemia  Acute, but ill-defined, cerebrovascular disease  Other and ill-defined cerebrovascular disease  Late effects of cerebrovascular disease | G45.x    G46.x    H34.0x  H34.1x  H34.2x  I60.x    I61.x    I62.x    I63.x  I64.x    I65.x      I66.x      I67.x  I68.x | Transient cerebral ischemic attacks and related syndromes  Vascular syndromes of brain in cerebrovascular diseases  Transient retinal artery occlusion  Central retinal artery occlusion  Other retinal artery occlusions  Non-traumatic subarachnoid hemorrhage  Non-traumatic intracerebral hemorrhage  Other and unspecified non-traumatic intracranial hemorrhage  Cerebral infarction  Stroke, not specified as hemorrhage or infarction  Occlusion and stenosis of precerebral arteries, not resulting in cerebral infarction  Occlusion and stenosis of cerebral arteries, not resulting in cerebral infarction  Other cerebrovascular diseases  Cerebrovascular disorders in diseases classified elsewhere |

*eTable 4e. Deyo and CDMF CCI Coding Schemes: Dementia*

| **ICD-9 Diagnosis Code** | | **ICD-10 Diagnosis Code** | |
| --- | --- | --- | --- |
| 290.0  290.1x  290.2x  290.3  290.4x  294.0  294.1x  294.2x  294.8  331.0  331.1x  331.2  331.7  797 | Senile dementia, uncomplicated  Presenile dementia  Senile dementia with delusional or depressive features  Senile dementia with delirium  Vascular dementia  Amnestic disorder in conditions classified elsewhere  Dementia in conditions classified elsewhere  Dementia, unspecified  Other persistent mental disorders due to conditions classified elsewhere  Alzheimer's disease  Frontotemporal dementia  Senile degeneration of brain  Cerebral degeneration in diseases classified elsewhere  Senility without mention of psychosis | F01.x  F02.x  F03.x  F04  F05  F06.1  F06.8  G13.2  G13.8  G30.x  G31.0x  G31.1  G31.2  G91.4  G94  R41.81  R54 | Vascular dementia  Dementia in other diseases classified elsewhere  Unspecified dementia  Amnestic disorder due to known physiological condition  Delirium due to known physiological condition  Catatonic disorder due to known physiological condition  Other specified mental disorders due to known physiological condition  Systemic atrophy primarily affecting the central nervous system in myxedema  Systemic atrophy primarily affecting central nervous system in other diseases classified elsewhere  Alzheimer's disease  Frontotemporal dementia  Senile degeneration of brain, not elsewhere classified  Degeneration of nervous system due to alcohol  Hydrocephalus in diseases classified elsewhere  Other disorders of brain in diseases classified elsewhere  Age-related cognitive decline  Age-related physical debility |

*eTable 4f. Deyo and CDMF CCI Coding Schemes: Chronic Pulmonary Disease*

| **ICD-9 Diagnosis Code** | | **ICD-10 Diagnosis Code** | |
| --- | --- | --- | --- |
| 490.x    491.x  492.x  493.x  494.x  495.x  496.x  500.x  501.x  502.x  503.x  504.x  505.x  506.4  508.1    508.8 | Bronchitis, not specified as acute or chronic  Chronic bronchitis  Emphysema  Asthma  Bronchiectasis  Extrinsic allergic alveolitis  Chronic airway obstruction, not elsewhere classified  Coal workers' pneumoconiosis  Asbestosis  Pneumoconiosis due to other silica or silicates  Pneumoconiosis due to other inorganic dust  Pneumonopathy due to inhalation of other dust  Pneumoconiosis, unspecified  Chronic respiratory conditions due to fumes and vapors  Chronic and other pulmonary manifestations due to radiation  Respiratory conditions due to other specified external agents | J40.x    J41.x    J42.x  J43.x  J44.x    J45.x  J46.x  J47.x  J60.x  J61.x    J62.x    J63.x    J64.x  J65.x    J66.x    J67.x    J68.4    J70.1    J70.3 | Bronchitis, not specified as acute or chronic  Simple and mucopurulent chronic bronchitis  Unspecified chronic bronchitis  Emphysema  Other chronic obstructive pulmonary disease  Asthma  Status asthmaticus  Bronchiectasis  Coal worker's pneumoconiosis  Pneumoconiosis due to asbestos and other mineral fibers  Pneumoconiosis due to dust containing silica  Pneumoconiosis due to other inorganic dusts  Unspecified pneumoconiosis  Pneumoconiosis associated with tuberculosis  Airway disease due to specific organic dust  Hypersensitivity pneumonitis due to organic dust  Chronic respiratory conditions due to chemicals, gases, fumes and vapors  Chronic and other pulmonary manifestations due to radiation  Chronic drug-induced interstitial lung disorders |

*eTable 4g. Deyo and CDMF CCI Coding Schemes: Rheumatic Disease*

| **ICD-9 Diagnosis Code** | | **ICD-10 Diagnosis Code** | |
| --- | --- | --- | --- |
| 446.5    710.0  710.1  710.2  710.3  710.4  714.0  714.1  714.2    714.8x    725.x | Polyarteritis nodosa and allied conditions  Systemic lupus erythematosus  Systemic sclerosis  Sicca syndrome  Dermatomyositis  Polymyositis  Rheumatoid arthritis  Felty's syndrome  Other rheumatoid arthritis with visceral or systemic involvement  Other specified inflammatory polyarthropathies  Polymyalgia rheumatica | M05.x    M06.x  M31.5    M32.x  M33.x  M34.x  M35.1  M35.3  M36.0 | Rheumatoid arthritis with rheumatoid factor  Other rheumatoid arthritis  Giant cell arteritis with polymyalgia rheumatica  Systemic lupus erythematosus (SLE)  Dermatopolymyositis  Systemic sclerosis [scleroderma]  Other overlap syndromes  Polymyalgia rheumatica  Dermato(poly)myositis in neoplastic disease |

*eTable 4h. Deyo and CDMF CCI Coding Schemes: Peptic Ulcer Disease*

| **ICD-9 Diagnosis Code** | | **ICD-10 Diagnosis Code** | |
| --- | --- | --- | --- |
| 531.x  532.x  533.x  534.x | Gastric ulcer  Duodenal ulcer  Peptic ulcer site unspecified  Gastrojejunal ulcer | K25.x  K26.x  K27.x  K28.x | Gastric ulcer  Duodenal ulcer  Peptic ulcer, site unspecified  Gastrojejunal ulcer |

*eTable 4i. Deyo and CDMF CCI Coding Schemes: Mild Liver Disease*

| **ICD-9 Diagnosis Code** | | **ICD-10 Diagnosis Code** | |
| --- | --- | --- | --- |
| 070.22    070.23    070.32      070.33      070.44  070.54    070.6    070.9    570.x  571.x  573.3  573.4  573.8  573.9  V42.7* | Chronic viral hepatitis B with hepatic coma without hepatitis delta  Chronic viral hepatitis B with hepatic coma with hepatitis delta  Chronic viral hepatitis B without mention of hepatic coma without mention of hepatitis delta  Chronic viral hepatitis B without mention of hepatic coma with hepatitis delta  Chronic hepatitis C with hepatic coma  Chronic hepatitis C without mention of hepatic coma  Unspecified viral hepatitis with hepatic coma  Unspecified viral hepatitis without mention of hepatic coma  Acute and subacute necrosis of liver  Chronic liver disease and cirrhosis  Hepatitis, unspecified  Hepatic infarction  Other specified disorders of liver  Unspecified disorder of liver  Liver replaced by transplant | B18.x  K70.0  K70.1  K70.2  K70.3  K70.9  K71.3    K71.4    K71.5    K71.7    K73.x    K74.x  K76.0    K76.2  K76.3  K76.4  K76.8  K76.9  Z94.4 | Chronic viral hepatitis  Alcoholic fatty liver  Alcoholic hepatitis  Alcoholic fibrosis and sclerosis of liver  Alcoholic cirrhosis of liver  Alcoholic liver disease, unspecified  Toxic liver disease with chronic persistent hepatitis  Toxic liver disease with chronic lobular hepatitis  Toxic liver disease with chronic active hepatitis  Toxic liver disease with fibrosis and cirrhosis of liver  Chronic hepatitis, not elsewhere classified  Fibrosis and cirrhosis of liver  Fatty (change of) liver, not elsewhere classified  Central hemorrhagic necrosis of liver  Infarction of liver  Peliosis hepatis  Other specified diseases of liver  Liver disease, unspecified  Liver transplant status |

*eTable 4j. Deyo and CDMF CCI Coding Schemes: Diabetes without chronic complication*

| **ICD-9 Diagnosis Code** | | **ICD-10 Diagnosis Code** | |
| --- | --- | --- | --- |
| 250.8x    250.9x  249.0x    249.1x    249.2x    249.3x    249.9x | Diabetes with other specified manifestations  Diabetes with unspecified complication  Secondary diabetes mellitus without mention of complication  Secondary diabetes mellitus with ketoacidosis  Secondary diabetes mellitus with hyperosmolarity  Secondary diabetes mellitus with other coma  Secondary diabetes mellitus with unspecified complication, no stated an uncontrolled, or unspecified | Main Codes  E08    E09    E10  E11  E13    Relevant Subcodes  E**.0x  E**.1x  E**.6x  E**.8x  E**.9x | Diabetes mellitus due to underlying condition  Drug or chemical induced diabetes mellitus  Type 1 diabetes mellitus  Type 2 diabetes mellitus  Other specified diabetes mellitus      With hyperosmolarity  With ketoacidosis  With other specified complications  With unspecified complications  Without complications |

*eTable 4k. Deyo and CDMF CCI Coding Schemes: Renal (Mild or Moderate)*

| **ICD-9 Diagnosis Code** | | **ICD-10 Diagnosis Code** | |
| --- | --- | --- | --- |
| 403.00      403.10      403.90      404.00        404.01        404.10        404.11        404.90        404.91        582.x  583.x  585.1  585.2  585.3    585.4  585.9  V42.0 | Hypertensive chronic kidney disease, malignant, with chronic kidney disease stage I through stage IV, or unspecified  Hypertensive chronic kidney disease, benign, with chronic kidney disease stage I through stage IV, or unspecified  Hypertensive chronic kidney disease, unspecified, with chronic kidney disease stage I through stage IV, or unspecified  Hypertensive heart and chronic kidney disease, malignant, without heart failure and with chronic kidney disease stage I through stage IV, or unspecified  Hypertensive heart and chronic kidney disease, malignant, with heart failure and with chronic kidney disease stage I through stage IV, or unspecified  Hypertensive heart and chronic kidney disease, benign, without heart failure and with chronic kidney disease stage I through stage IV, or unspecified  Hypertensive heart and chronic kidney disease, benign, with heart failure and with chronic kidney disease stage I through stage IV, or unspecified  Hypertensive heart and chronic kidney disease, unspecified, without heart failure and with chronic kidney disease stage I through stage IV, or unspecified  Hypertensive heart and chronic kidney disease, unspecified, with heart failure and with chronic kidney disease stage I through stage IV, or unspecified  Chronic glomerulonephritis  Nephritis/nephropathy  Chronic kidney disease, Stage I  Chronic kidney disease, Stage II (mild)  Chronic kidney disease, Stage III (moderate)  Chronic kidney disease, Stage IV (severe)  Chronic kidney disease, unspecified  Kidney replaced by transplant | I12.9        I13.0        I13.10        N03.x  N05.x  N18.1  N18.2  N18.3    N18.4  N18.9  Z94.0 | Hypertensive chronic kidney disease with stage 1 through stage 4 chronic kidney disease, or unspecified chronic kidney disease  Hypertensive heart and chronic kidney disease with heart failure and stage 1 through stage 4 chronic kidney disease, or unspecified chronic kidney disease  Hypertensive heart and CKD without heart failure with stage 1 through stage 4 chronic kidney disease, or unspecified chronic kidney disease  Chronic nephritic syndrome  Unspecified nephritic syndrome  Chronic kidney disease, Stage 1  Chronic kidney disease, Stage 2 (mild)  Chronic kidney disease, Stage 3 (moderate)  Chronic kidney disease, Stage 4 (severe)  Chronic kidney disease, unspecified  Kidney transplant status |

NOTE: Deyo et al. (1992) used one set of codes without differentiation by severity: 582.x, 583.0, 583.1, 583.2, 583.3, 583.4, 583.5, 583.6, 583.7, 585.x, 586.x, and 588.x.

*eTable 4l. Deyo and CDMF CCI Coding Schemes: Diabetes with Chronic Complications*

| **ICD-9 Diagnosis Code** | | **ICD-10 Diagnosis Code** | |
| --- | --- | --- | --- |
| 250.4  250.5    250.6    250.7 | Diabetes with renal manifestations  Diabetes with ophthalmic complications  Diabetes with neurological manifestations  Diabetes with peripheral circulatory disorders | Main Codes  E08    E09    E10  E11  E13    Relevant Subcodes  E**.2    E**.3      E**.4      E**.5 | Diabetes mellitus due to underlying condition  Drug or chemical induced diabetes mellitus  Type 1 diabetes mellitus  Type 2 diabetes mellitus  Other specified diabetes mellitus      Diabetes mellitus due to underlying condition with kidney complications  Diabetes mellitus due to underlying condition with ophthalmic complications  Diabetes mellitus due to underlying condition with neurological complications  Diabetes mellitus due to underlying condition with circulatory complications |

*eTable 4m. Deyo and CDMF CCI Coding Schemes: Hemiplegia or Paraplegia*

| **ICD-9 Diagnosis Code** | | **ICD-10 Diagnosis Code** | |
| --- | --- | --- | --- |
| 334.1  342.x  343.x  344.x | Hereditary spastic paraplegia  Hemiplegia and hemiparesis  Infantile cerebral palsy  Other paralytic syndromes | G04.1  G11.4  G80.0  G80.1  G80.2  G81.x  G82.x    G83.x | Tropical spastic paraplegia  Hereditary spastic paraplegia  Spastic quadriplegic cerebral palsy  Spastic diplegic cerebral palsy  Spastic hemiplegic cerebral palsy  Hemiplegia and hemiparesis  Paraplegia (paraparesis) and quadriplegia (quadriparesis)  Other paralytic syndromes |

*eTable 4n. Deyo and CDMF CCI Coding Schemes: Any malignancy, including lymphoma and leukemia, except malignant nonmelanoma neoplasm of skin*

| **ICD-9 Diagnosis Code** | | **ICD-10 Diagnosis Code** | |
| --- | --- | --- | --- |
| 14x.x    15x.x    16x.x    170.x    171.x    172.x  174.x  175.x  176.x  179.x    18x.x    190.x  191.x  192.x    193.x  194.x    195.x    199.1    200.x      201.x  202.x    203.x    204.x  205.x  206.x  207.x  208.x  238.6 | Malignant neoplasm of lip, oral cavity, and pharynx  Malignant neoplasm of digestive organs and peritoneum  Malignant neoplasm of respiratory and intrathoracic organs  Malignant neoplasm of bone and articular cartilage  Malignant neoplasm of connective and other soft tissue  Malignant melanoma of skin  Malignant neoplasm of female breast  Malignant neoplasm of male breast  Kaposi's sarcoma  Malignant neoplasm of uterus, part unspecified  Malignant neoplasm of genitourinary organs  Malignant neoplasm of eye  Malignant neoplasm of brain  Malignant neoplasm of other and unspecified parts of nervous system  Malignant neoplasm of thyroid gland  Malignant neoplasm of other endocrine glands and related structures  Malignant neoplasm of other and ill-defined sites  Other malignant neoplasm without specification of site  Lymphosarcoma and reticulosarcoma and other specified malignant tumors of lymphatic tissue  Hodgkin's disease  Other malignant neoplasms of lymphoid and histiocytic tissue  Multiple myeloma and immunoproliferative neoplasms  Lymphoid leukemia  Myeloid leukemia  Monocytic leukemia  Other specified leukemia  Leukemia of unspecified cell type  Neoplasm of uncertain behavior of plasma cells | C0x.x    C1x.x      C2x.x            C30.x    C31.x    C32.x  C33.x  C34.x    C37.x  C38.x    C39.x      C40.x    C41.x      C43.x  C45.x  C46.x  C47.x    C48.x    C49.x    C50  C51-58.x  C60-63.x  C76.x    C80.1    C81.x  C82.x  C83.x  C84.x  C85.x  C88.x  C9x.x | Malignant neoplasms of lip, oral cavity and pharynx  Malignant neoplasms of lip, oral cavity and pharynx, esophagus, stomach, small intestine, colon  Malignant neoplasms of rectum, anus, anal canal, liver and intrahepatic bile ducts, gallbladder, other and unspecified part of biliary tract, pancreas, other and ill-defined digestive organs  Malignant neoplasm of nasal cavity and middle ear  Malignant neoplasm of accessory sinuses  Malignant neoplasm of larynx  Malignant neoplasm of trachea  Malignant neoplasm of bronchus and lung  Malignant neoplasm of thymus  Malignant neoplasm of heart, mediastinum and pleura  Malignant neoplasm of other and ill-defined sites in the respiratory system and intrathoracic organs  Malignant neoplasm of bone and articular cartilage of limbs  Malignant neoplasm of bone and articular cartilage of other and unspecified sites  Malignant melanoma of skin  Mesothelioma  Kaposi's sarcoma  Malignant neoplasm of peripheral nerves and autonomic nervous system  Malignant neoplasm of retroperitoneum and peritoneum  Malignant neoplasm of other connective and soft tissue  Malignant neoplasm of breast  Malignant neoplasms of female genital organs  Malignant neoplasm of male genital organs  Malignant neoplasm of other and ill-defined sites  Malignant (primary) neoplasm, unspecified  Hodgkin lymphoma  Follicular lymphoma  Non-follicular lymphoma  Mature T/NK-cell lymphomas  Other specified and unspecified types of non-Hodgkin lymphoma  Malignant immunoproliferative diseases and certain other B-cell lymphomas  Multiple myeloma and malignant plasma cell neoplasms, lymphoid leukemia, monocytic leukemia, other leukemias of specified cell type, leukemia of unspecified cell type, other and unspecified malignant neoplasms of lymphoid, hematopoietic and related tissue |

*eTable 4o. Deyo and CDMF CCI Coding Schemes: Moderate or Severe Liver Disease*

| **ICD-9 Diagnosis Code** | | **ICD-10 Diagnosis Code** | |
| --- | --- | --- | --- |
| 456.0  456.1    456.2x    572.2  572.3  572.4  572.8 | Esophageal varices with bleeding  Esophageal varices without mention of bleeding  Esophageal varices in diseases classified elsewhere  Hepatic encephalopathy  Portal hypertension  Hepatorenal syndrome  Other sequelae of chronic liver disease | I85.0x  I86.4  K70.4x  K71.1x    K72.1x  K72.9x  K76.5  K76.6  K76.7 | Esophageal varices  Gastric varices  Alcoholic hepatic failure  Toxic liver disease with hepatic necrosis  Chronic hepatic failure  Hepatic failure, unspecified  Hepatic veno-occlusive disease  Portal hypertension  Hepatorenal syndrome |

*eTable 4p. Deyo and CDMF CCI Coding Schemes: Renal (Severe)*

| **ICD-9 Diagnosis Code** | | **ICD-10 Diagnosis Code** | |
| --- | --- | --- | --- |
| 403.01      403.11      403.91      404.02        404.03        404.12        404.13        404.92        404.93        585.5  585.6  586.x  588.0  V45.11  V45.12  V56.0  V56.1    V56.2    V56.31    V56.32    V56.8 | Hypertensive chronic kidney disease, malignant, with chronic kidney disease stage V or end stage renal disease  Hypertensive chronic kidney disease, benign, with chronic kidney disease stage V or end stage renal disease  Hypertensive chronic kidney disease, unspecified, with chronic kidney disease stage V or end stage renal disease  Hypertensive heart and chronic kidney disease, malignant, without heart failure and with chronic kidney disease stage V or end stage renal disease  Hypertensive heart and chronic kidney disease, malignant, with heart failure and with chronic kidney disease stage V or end stage renal disease  Hypertensive heart and chronic kidney disease, benign, without heart failure and with chronic kidney disease stage V or end stage renal disease  Hypertensive heart and chronic kidney disease, benign, with heart failure and chronic kidney disease stage V or end stage renal disease  Hypertensive heart and chronic kidney disease, unspecified, without heart failure and with chronic kidney disease stage V or end stage renal disease  Hypertensive heart and chronic kidney disease, unspecified, with heart failure and chronic kidney disease stage V or end stage renal disease  Chronic kidney disease, Stage V  End stage renal disease  Renal failure NOS  Renal osteodystrophy  Renal dialysis status  Noncompliance with renal dialysis  Encounter for extracorporeal dialysis  Fitting and adjustment of extracorporeal dialysis catheter  Fitting and adjustment of peritoneal dialysis catheter  Encounter for adequacy testing for hemodialysis  Encounter for adequacy testing for peritoneal dialysis  Encounter for other dialysis | I12.0      I13.11      I13.2        N18.5  N18.6  N19.x  N25.0  Z49.x  Z99.2 | Hypertensive chronic kidney disease with stage 5 chronic kidney disease or end stage renal disease  Hypertensive heart and CKD without heart failure with stage 5 chronic kidney disease, or end stage renal disease  Hypertensive heart and chronic kidney disease with heart failure and with stage 5 chronic kidney disease, or end stage renal disease  Chronic kidney disease, Stage 5  End stage renal disease  Unspecified kidney failure  Renal osteodystrophy  Encounter for care involving renal dialysis  Dependence on renal dialysis |

NOTE: Deyo et al. (1992) used one set of codes without differentiation by severity: 582.x, 583.0, 583.1, 583.2, 583.3, 583.4, 583.5, 583.6, 583.7, 585.x, 586.x, and 588.x.

*eTable 4q. Deyo and CDMF CCI Coding Schemes: HIV Infection*

| **ICD-9 Diagnosis Code** | | **ICD-10 Diagnosis Code** | |
| --- | --- | --- | --- |
| 042.x | Human immunodeficiency virus [HIV] disease | B20.x | Human immunodeficiency virus [HIV] disease |

NOTE: The Deyo version of the ICD-9 coding system includes codes 043.x and 044.x, which are no longer in use. Corresponding ICD-10 codes B21.x, B22.x, and B24.x also no longer exist.

*eTable 4r. Deyo and CDMF CCI Coding Schemes: Metastatic Solid Tumor*

| **ICD-9 Diagnosis Code** | | **ICD-10 Diagnosis Code** | |
| --- | --- | --- | --- |
| 196.x    197.x    198.x    199.0 | Secondary and unspecified malignant neoplasm of lymph nodes  Secondary malignant neoplasm of respiratory and digestive systems  Secondary malignant neoplasm of other specified sites  Disseminated malignant neoplasm without specification of site | C77.x    C78.x    C79.x    C80.0    C80.2 | Secondary and unspecified malignant neoplasm of lymph nodes  Secondary malignant neoplasm of respiratory and digestive organs  Secondary malignant neoplasm of other and unspecified sites  Disseminated malignant neoplasm, unspecified  Malignant neoplasm associated with transplanted organ |

*eTable 4s. Deyo and CDMF CCI Coding Schemes: : AIDS (HIV Infection + opportunistic infection)*

| **ICD-9 Diagnosis Code** | | **ICD-10 Diagnosis Code** | |
| --- | --- | --- | --- |
| 112.x    180.x  114.x  117.5  007.4    078.5    348.3x  054.x      115.x  007.2    176.x  200-209  031.x  010-018  136.3  V12.61  046.3    003.1  130.x  799.4 | Candidiasis of bronchi, trachea, esophagus, or lungs  Invasive cervical cancer  Coccidioidomycosis  Cryptococcosis  Cryptosporidiosis, chronic intestinal (greater than 1 month's duration)  Cytomegalovirus disease (particularly CMV retinitis)  Encephalopathy, HIV-related  Herpes simplex: chronic ulcer(s) (greater than 1 month's duration); or bronchitis, pneumonitis, or esophagitis  Histoplasmosis  Isosporiasis, chronic intestinal (greater than 1 month's duration)  Kaposi's sarcoma  Lymphoma, multiple forms  Mycobacterium avium comp  Tuberculosis  Pneumocystis carinii pneumonia  Pneumonia, recurrent  Progressive multifocal leukoencephalopathy  Salmonella septicemia, recurrent  Toxoplasmosis of brain  Wasting syndrome due to HIV | B37.x      C53.x  B38.x  B45.x  A07.2    B25.x  G93.4x  B00  B39.x  A07.3  C46.x  C81-C96  A31.x  A15-A19  B59  Z87.01  A81.2  A02.1  B58.x  R64 | Candidiasis of bronchi, trachea, esophagus, or lungs  Invasive cervical cancer  Coccidioidomycosis  Cryptococcosis  Cryptosporidiosis, chronic intestinal (greater than 1 month's duration)  Cytomegalovirus disease (particularly CMV retinitis)  Encephalopathy, HIV-related  Herpes simplex: chronic ulcer(s) (greater than 1 month's duration); or bronchitis, pneumonitis, or esophagitis  Histoplasmosis  Isosporiasis, chronic intestinal (greater than 1 month's duration)  Kaposi's sarcoma  Lymphoma, multiple forms  Mycobacterium avium comp  Tuberculosis  Pneumocystis carinii pneumonia  Pneumonia, recurrent  Progressive multifocal leukoencephalopathy  Salmonella septicemia, recurrent  Toxoplasmosis of brain  Wasting syndrome due to HIV |

# eTable 5: Code sets for SDoH.

| **Phenotype** | **Code Type** | **Concept ID** | **Concept Name** |
| --- | --- | --- | --- |
| **Disabilities** |  |  |  |
| **Hearing** | OMOP, Question | 903573 | Disability: Deaf (Are you deaf, or do you have serious difficulty hearing?) |
|  | OMOP, Answer | 903503 | Deaf: No |
|  | OMOP, Answer | 903587 | Deaf: Yes |
|  | OMOP, Answer | 903096 | PMI: Skip |
|  | OMOP, Answer | 903596 | Deaf: Prefer Not To Answer |
| **Vision** | OMOP, Question | 903574 | Disability: Blind (Are you blind, or do you have serious difficulty seeing, even when wearing glasses?) |
|  | OMOP, Answer | 903597 | Blind: No |
|  | OMOP, Answer | 903504 | Blind: Yes |
|  | OMOP, Answer | 903096 | PMI: Skip |
|  | OMOP, Answer | 903598 | Blind: Prefer Not To Answer |
| **Cognition** | OMOP, Question | 903575 | Disability: Difficulty Concentrating (Because of a physical, mental, or emotional condition, do you have serious difficulty concentrating, remembering, or making decisions?) |
|  | OMOP, Answer | 903600 | Difficulty Concentrating: No |
|  | OMOP, Answer | 903599 | Difficulty Concentrating: Yes |
|  | OMOP, Answer | 903096 | PMI: Skip |
|  | OMOP, Answer | 903601 | Difficulty Concentrating: Prefer Not To Answer |
| **Mobility** | OMOP, Question | 903576 | Disability: Walking Climbing (Do you have serious difficulty walking or climbing stairs?) |
|  | OMOP, Answer | 903603 | Walking Climbing: No |
|  | OMOP, Answer | 903602 | Walking Climbing: Yes |
|  | OMOP, Answer | 903096 | PMI: Skip |
|  | OMOP, Answer | 903604 | Walking Climbing: Prefer Not To Answer |
| **Self-care** | OMOP, Question | 903577 | Disability: Dressing Bathing (Do you have difficulty dressing or bathing?) |
|  | OMOP, Answer | 903606 | Dressing Bathing: No |
|  | OMOP, Answer | 903605 | Dressing Bathing: Yes |
|  | OMOP, Answer | 903096 | PMI: Skip |
|  | OMOP, Answer | 903607 | Dressing Bathing: Prefer Not To Answer |
| **Independent Living** | OMOP, Question | 903578 | Disability: Errands Alone (Because of a physical, mental, or emotional condition, do you have difficulty doing errands alone such as visiting a physician's office or shopping?) |
|  | OMOP, Answer | 903609 | Errands Alone: No |
|  | OMOP, Answer | 903608 | Errands Alone: Yes |
|  | OMOP, Answer | 903096 | PMI: Skip |
|  | OMOP, Answer | 903610 | Errands Alone: Prefer Not To Answer |
| **Health Insurance** | OMOP, Question | 1585389 | Insurance: Health Insurance (Are you covered by health insurance or some other kind of health care plan?) |
|  | OMOP, Answer | 1585387 | Health Insurance: Yes |
|  | OMOP, Answer | 1585388 | Health Insurance: No |
|  | OMOP, Answer | 903087 | PMI: Dont Know |
|  | OMOP, Answer | 903079 | PMI: Prefer Not To Answer |
|  | OMOP, Answer | 903096 | PMI: Skip |
|  | OMOP, Question | 43528428 | Health Insurance: Insurance Type Update (Are you currently covered by any of the following types of health insurance or health coverage plans? Select all that apply from one group) |
|  | OMOP, Answer | 43529120 | Insurance Type Update: Employer Or Union |
|  | OMOP, Answer | 43529210 | Insurance Type Update: Medicare |
|  | OMOP, Answer | 43529209 | Insurance Type Update: Medicaid |
|  | OMOP, Answer | 43529119 | Insurance Type Update: Purchased |
|  | OMOP, Answer | 43529926 | Insurance Type Update: VA |
|  | OMOP, Answer | 43528423 | Insurance Type Update: Other Health Plan |
|  | OMOP, Answer | 43529920 | Insurance Type Update: Military |
|  | OMOP, Answer | 903096 | PMI: Skip |
|  | OMOP, Answer | 43529095 | Insurance Type Update: None |
|  | OMOP, Answer | 43529111 | Insurance Type Update: Indian |
| **Employment** | OMOP, Question | 1585952 | Employment: Employment Status (What is your current employment status? Please select 1 or more of these categories) |
|  | OMOP, Answer | 1585955 | Employment Status: Out Of Work One Or More |
|  | OMOP, Answer | 1585956 | Employment Status: Out Of Work Less Than One |
|  | OMOP, Answer | 903079 | PMI: Prefer Not To Answer |
|  | OMOP, Answer | 1585957 | Employment Status: Homemaker |
|  | OMOP, Answer | 1585954 | Employment Status: Self Employed |
|  | OMOP, Answer | 903096 | PMI: Skip |
|  | OMOP, Answer | 1585959 | Employment Status: Retired |
|  | OMOP, Answer | 1585960 | Employment Status: Unable To Work |
|  | OMOP, Answer | 1585958 | Employment Status: Student |
|  | OMOP, Answer | 1585953 | Employment Status: Employed For Wages |
| **Income** | OMOP, Question | 1585375 | Income: Annual Income (What is your annual household income from all sources?) |
|  | OMOP, Answer | 1585380 | Annual Income: 50k 75k |
|  | OMOP, Answer | 1585378 | Annual Income: 25k 35k |
|  | OMOP, Answer | 1585379 | Annual Income: 35k 50k |
|  | OMOP, Answer | 903079 | PMI: Prefer Not To Answer |
|  | OMOP, Answer | 1585376 | Annual Income: less 10k |
|  | OMOP, Answer | 1585383 | Annual Income: 150k 200k |
|  | OMOP, Answer | 1585384 | Annual Income: more 200k |
|  | OMOP, Answer | 1585377 | Annual Income: 10k 25k |
|  | OMOP, Answer | 1585381 | Annual Income: 75k 100k |
|  | OMOP, Answer | 903096 | PMI: Skip |
|  | OMOP, Answer | 1585382 | Annual Income: 100k 150k |
| **Education** | OMOP, Question | 1585940 | Education Level: Highest Grade (What is the highest grade or year of school you completed?) |
|  | OMOP, Answer | 1585941 | Highest Grade: Never Attended |
|  | OMOP, Answer | 1585945 | Highest Grade: Twelve Or GED |
|  | OMOP, Answer | 1585942 | Highest Grade: One Through Four |
|  | OMOP, Answer | 1585947 | Highest Grade: College Graduate |
|  | OMOP, Answer | 1585946 | Highest Grade: College One to Three |
|  | OMOP, Answer | 1585944 | Highest Grade: Nine Through Eleven |
|  | OMOP, Answer | 1585948 | Highest Grade: Advanced Degree |
|  | OMOP, Answer | 1585943 | Highest Grade: Five Through Eight |
|  | OMOP, Answer | 903096 | PMI: Skip |
|  | OMOP, Answer | 903079 | PMI: Prefer Not To Answer |
| **Housing** | OMOP, Question | 1585370 | Home Own: Current Home Own |
|  | OMOP, Answer | 1585372 | Current Home Own: Rent |
|  | OMOP, Answer | 1585371 | Current Home Own: Own |
|  | OMOP, Answer | 1585373 | Current Home Own: Other Arrangement |
|  | OMOP, Answer | 903096 | PMI: Skip |
|  | OMOP, Answer | 903087 | PMI: Dont Know |
|  | OMOP, Answer | 903079 | PMI: Prefer Not To Answer |
| **Housing Stability** | OMOP, Question | 1585886 | Living Situation: Stable House Concern (In the past 6 months, have you been worried or concerned about NOT having a place to live?) |
|  | OMOP, Answer | 1585887 | Stable House Concern: Yes |
|  | OMOP, Answer | 1585888 | Stable House Concern: No |
|  | OMOP, Answer | 903096 | PMI: Skip |

# eTable 6: Acidosis-related genotypes.

| **Chr:Locus** | **Genotype** | **Gene** | **Function** | **Ref** |
| --- | --- | --- | --- | --- |
| **17:19560030** | G>A | SLC47A1 | It encodes a protein of unknown function | rs2289669 |
| **17:19561878** | C>T | SLC47A1 | It encodes a protein of unknown function | rs8065082 |
| **17:19716685** | G>A | SLC47A2 | Transporters involved in excretion of toxic electrolytes, both endogenous and exogenous, through urine and bile. | rs12943590 |
| **6:160122116** | C>T | SLC22A1 | Polyspecific organic cation transporters in the liver, kidney, intestine, and other organs | rs12208357 |
| **6:160139849-160139853** | delGAT | SLC22A1 | Polyspecific organic cation transporters in the liver, kidney, intestine, and other organs |  |
| **6:160151834** | C>A / C>G | SLC22A1 | Polyspecific organic cation transporters in the liver, kidney, intestine, and other organs | rs622342 |
| **6:160249250** | A>C / A>T | SLC22A2 | Polyspecific organic cation transporters in the liver, kidney, intestine, and other organs | rs316019 |
| **5:132327369** | T>C / T>G | SLC22A4 | Polyspecific organic cation transporters in the liver, kidney, intestine, and other organs | rs272893 |

# eTable 7: Sex Assigned at Birth and Gender Identity Concordance.

|  | **Gender: Man** | **Gender: Woman** | **Gender: Other** |
| --- | --- | --- | --- |
| **Sex: Male** | 4602 | <20 | <70 |
| **Sex: Female** | <20 | 8191 | <130 |
| **Sex: Other** | <70 | <90 | 177 |

# eMethod

**Missing data processing.**

There are three types of missingness in this multi-domain data: domain-level missing, longitudinal-level missing, and data-element-level missing. We comprehensively handled missing data as:

1. If the entire data domain is missing, the participants are excluded from the study. As shown in Supplement eFigure 1, 182,497 participants were excluded because the diagnosis data domain or the basic survey data domain was missing.
2. For longitudinal data, even if a data domain is available, it is common in real-world data that some longitudinal records are missing. The potential biases due to longitudinal data incompleteness were handled by the propensity score matching for the number of diagnosis records and the length of medical history (eFigure 1).
3. The missing data in a specific data element was labeled as a categorical value called “Missing” and was used in the analysis. We assumed informative missingness. That is, the pattern of missing data reflects independent information related to participants’ health. Therefore, the missing data should not be imputed from available data, and participants with missing data should not be excluded from the analysis. The advantages of treating missing data as a category are: 1) Avoid bias in cohorts due to missing data in SDoH. Since no patient was excluded from the analysis, all SDoH analyses were on the same cohort; 2) Adjust the analysis for the informative missingness. For example, in the analysis of education level, 3.4% of participants (453 out of 13,330) did not have the data and thus were labeled as “Missing”. The corresponding adjusted OR and P-value of this group compared with the reference group (College Graduate or Advanced) were 1.24 and 0.23, respectively.

**Sample balancing.**

As shown in **eTable 8**, there were significant differences in the length of the medical history and the number of diagnoses between patients with severe acidosis after enrollment and patients without any acidosis records. Those who did not have acidosis records had shorter medical histories (median start dates of 6/12/2013 vs 5/16/2009 or 5.62 years shorter). Consistently, the median number of diagnosis records is less (30 vs 134). The standardized differences of these two features are 1.19 and 1.89, respectively. The significantly lower longitudinal completeness of the non-acidosis patient group may cause under-reported clinical conditions such as comorbidities as well as other confounding effects. After propensity score matching (**eTable 9**), the case and the control groups have similar longitudinal data completeness, with standardized differences between 0.109 – 0.196. The potential biases due to longitudinal data completeness were mitigated.

# eTable 8: Pre-matching sample balance table

| **Variable**  **median (IQR)** | **Severe acidosis**  **(N = 2,676)** | **No acidosis record**  **(N = 251,317)** | **Standardized difference** |
| --- | --- | --- | --- |
| **Enrollment date** | 4/29/2019  (9/28/2018 – 11/14/2019) | 7/9/2019  (11/14/2018 – 2/17/2020) | -0.210 |
| **Start of medical history** | 5/16/2009  (11/22/2002 – 1/16/2014) | 6/12/2013  (6/12/2013 – 9/8/2016) | -1.19 |
| **Number of diagnoses** | 134 (74 – 215) | 30 (10 – 73) | 1.89 |

# eTable 9: Post-matching sample balance table

| **Variable**  **(median and IQR)** | **Case group**  **(N = 2,666)** | **Control group**  **(N = 10,664)** | **Standardized difference** |
| --- | --- | --- | --- |
| **Enrollment date** | 4/29/2019  (9/28/2018 – 11/14/2019) | 2/26/2019  (7/20/2018 – 9/23/2019) | 0.196 |
| **Start of medical history** | 5/16/2009  (11/22/2002 – 1/16/2014) | 9/20/2007  (10/5/1999 – 7/17/2013) | 0.168 |
| **Number of diagnoses** | 134 (74 – 215) | 121 (50 – 216) | 0.109 |

# eFigure 2. Longitudinal availability of diagnosis data in the case and the control groups


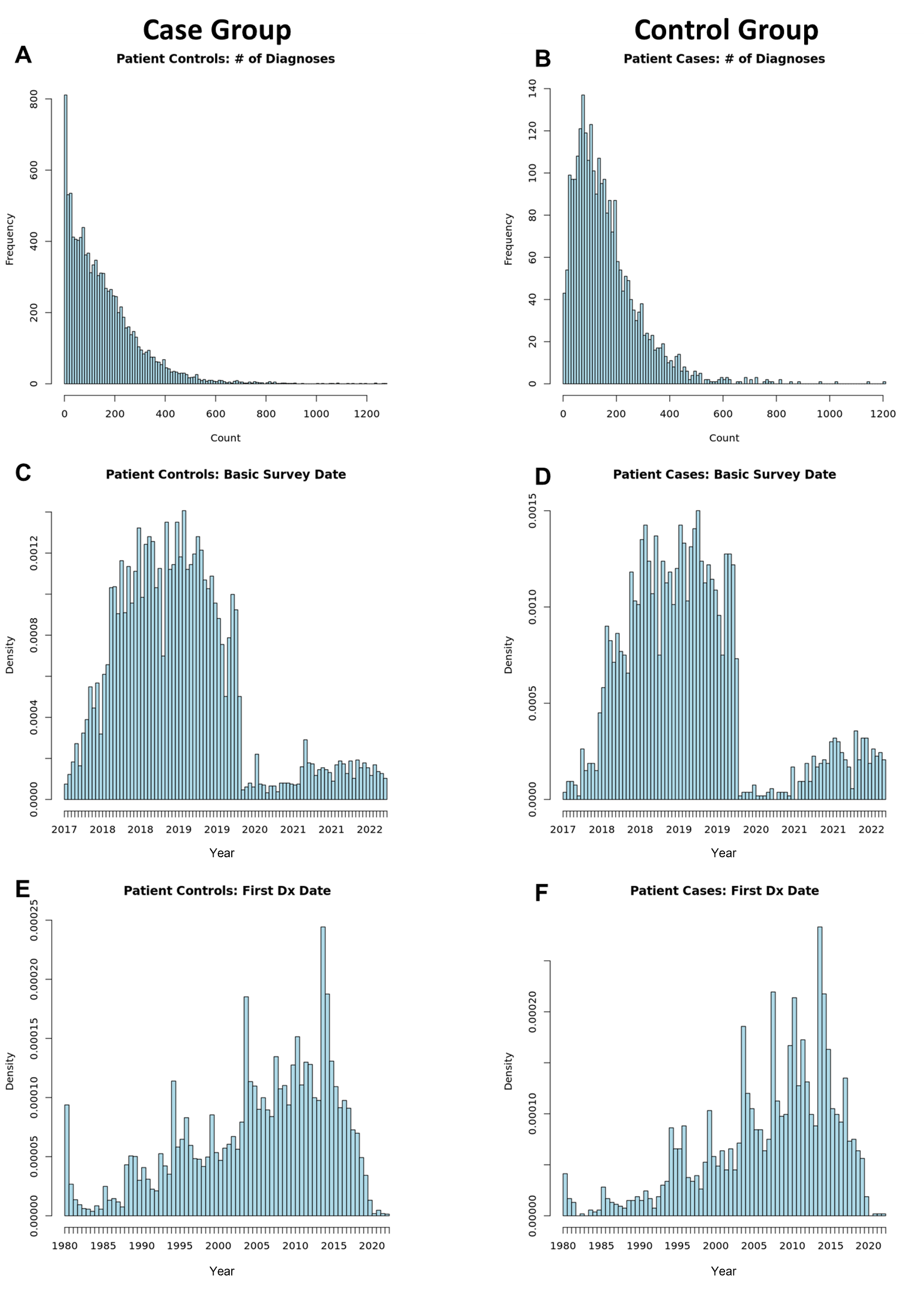


# eFigure 3. Adjusted odds ratios of the base model.


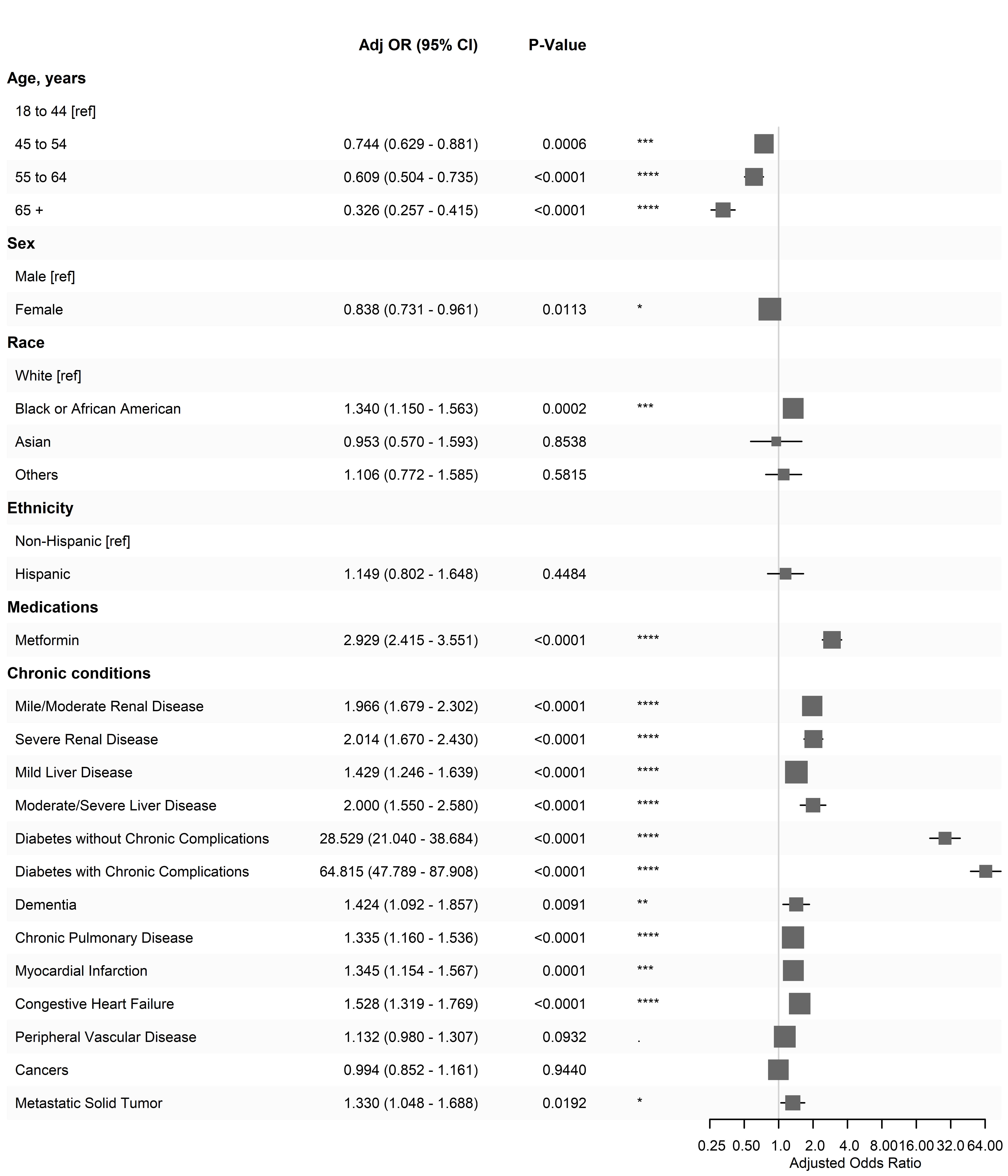

Supplement: ocae256_Supplementary_Data [file ocae256_supplementary_data.docx]
